# Transcriptome alteration spectrum in rat lung induced by radiotherapy

**DOI:** 10.1038/s41598-019-56027-4

**Published:** 2019-12-23

**Authors:** Tao Zhang, Guowei Cheng, Li Sun, Lei Deng, Xin Wang, Nan Bi

**Affiliations:** 1 Department of Radiation Oncology, National Cancer Center/National Clinical Research Center for Cancer/Cancer Hospital, Chinese Academy of Medical Science, Peking Union Medical College, Beijing, 100021 China; 2 Department of Radiation Oncology, Cancer Hospital of HuanXing ChaoYang District Beijing, Beijing, 100122 China

**Keywords:** Gene expression, Long non-coding RNAs

## Abstract

Radiation therapy is crucial for curative treatment of lung cancer, which frequently leads to lung injury. Long non-coding RNAs (lncRNAs) are a group of RNAs longer than 200 nucleotides and lack protein-coding capacity. Increasing evidences demonstrate the important roles of lncRNAs in biological processes. However, the mechanism underlying the association of ionizing radiation with alterations in mRNA and lncRNA expression and lung injury remains unclear. In our study, the male Sprague-Dawley (SD) rats were exposed to a dose of 18 Gy of 6 MV X-ray and the transcriptome spectrum was studied. To identify the differentially expressed mRNAs and lncRNAs induced by X-ray, the RNA sequencing data of lung tissues from irradiated and normal rats for 4, 8, and 16 weeks were analyzed, using |log2_ratio| ≥ 1 and q ≤ 0.05 as thresholds for significantly differential expression. The number of differentially expressed mRNAs was 1097 (686 up- and 411 down-) for 4-week radiotherapy group, 3006 (1935 up- and 1071 down-) for 8-week group and 1838 (1178 up- and 660 down-) for 16-week group. There were 606 (279 up- and 327 down-) differentially expressed lncRNAs in 4-week group, 1715 (831 up- and 884 down-) in 8-week group and 1043 (656 up- and 387 down-) in 16-week group. The differentially expressed mRNAs were mainly involved in cell cycle regulation and Fc receptor pathway, while the lncRNA target genes were significantly enriched in cellular stress response and regulation of cell migration. Moreover, compared with the control group, the irradiated group presented higher tissue specificity of lncRNAs. Radiation-induced lung injury, especially the dynamic network of lncRNAs and mRNAs, is worthy of study. Investigation on the regulatory details of related pathways is significant for the prevention of radiation-related lung injury, as well as the improvement of radiation therapy.

## Introduction

Radiation therapy is a common and efficient treatment for lung cancer, improving patients’ survival^1,2^. However, radiation therapy also causes strong side effects in patients such as acute pneumonitis and chronic fibrosis, both of which are fatal^3^. The side effects of radiation therapy on patients are mainly due to the reactive damages of various cellular components induced by ionizing radiation^4,5^. Previous studies have found that radiation therapy induces DNA double-strand breaks and other DNA damages, activation of DNA repair reactions and other cellular pathways, such as unfolded protein response (UPR) or autophagy^6,7^. Activation of these signaling pathways leads to reprogramming of the cellular transcriptional network to restore the structure of the DNA and eliminate damaged cellular components. Investigation of molecular mechanisms underlying radiation therapy damages will help develop new strategies to reduce the side effects for patients with lung cancer.

Previous genetic studies mostly focused on protein-coding RNAs, which account for only 2% of genome transcripts^8–11^. For example, cytokines have been proved to play a pivotal role in lung injury induced by radiation and alterations in cytokines levels have been observed both *in vitro* and *in vivo*^12–14^. In comparison to the control group, the TGF-β1 level was significantly elevated over time in the lung tissues of irradiated rats^15^. Obvious difference in angiotensin II expression level was also observed in the lung tissues between irradiated and non-irradiated rats^15^. Moreover, aberrant expressions, such as sphingolipid metabolic pathway-related genes, p53, and nuclear factor-erythroid-2-related factor, were implicated in radiation induced-lung injury^16^. These studies suggest that the corresponding mRNAs related to above genes or proteins may act as potentially biomarkers in radiation induced-lung injury. In addition, non-coding RNAs, which were once considered as transcriptional noise, are proved to be implicated multiple physiological and pathological processes, such as tumor development^17–19^. Long non-coding RNAs (lncRNAs) refer to a group of RNAs longer than 200 nucleotides and lacking protein-coding ability. Due to their low abundance, interspecific conservation and high tissue specificity, the researches on lncRNAs, especially on the functional annotations are relatively limited^10,20–22^. An increasing number of studies have revealed that lncRNA is closely involved in various biological processes, including remodeling of chromatin, miRNA sponges, and epigenetic modification^11,22–24^. So, identifying more novel lncRNAs that play important roles in tumor radiotherapy should be helpful for ameliorating the survival of patients after radiation.

Herein, we aimed to investigate the effects of radiation therapy on mRNA and lncRNA expression profiles in rats during the early stage of lung injury induced by radiation, using high-throughput sequencing. As Kampinga HH *et al*. have shown that Sprague-Dawley rats presented an elevated breathing rate starting 4 weeks after irradiation^25^, we adopted the Sprague-Dawley rats and selected three time points of 4 weeks, 8 weeks, and 16 weeks to study the transcriptome alterations during the early stage of lung injury induced by radiation. A cohort of differentially expressed mRNAs and lncRNAs induced by radiation in rats’ lung tissues were identified. Functional analysis suggested that differentially expressed mRNAs and lncRNAs were significantly implicated in DNA replicate and cell apoptosis signaling pathways. Moreover, lncRNAs exhibited a higher tissue specificity than mRNAs, which implicated a crucial function of lncRNA in radiotherapy. This study will help identify novel lncRNAs that have important functions in radiation therapy.

## Methods and Materials

### Study subjects

Animal experiments were granted an exemption from requiring ethics approval with National Cancer Center/National Clinical Research Center for Cancer/Cancer Hospital, Chinese Academy of Medical Science, Peking Union Medical College. Male Sprague-Dawley (SD) rats (4 weeks old) were purchased from the Shanghai SLAC Laboratory Animal Co., Ltd. (Shanghai, China). The animals were housed with a 12-h light/dark cycle with food and water ad libitum. After anesthetized with intra-peritoneally administered 5% Pentobarbital sodium injection, the rats were fixed during radiation exposure. The rats in control group were treated using the same manner with those in experimental group except for the irradiation. The clinical samples were divided into three groups: radiotherapy for 4 weeks (n_Con_ = 4, n_exp_ = 5); radiotherapy for 8 weeks (n_Con_ = 5, n_exp_ = 4); radiotherapy for 16 weeks (n_Con_ = 5, n_exp_ = 5). n_Con_ represented the number of rats in control group and n_exp_ represented that in experimental group.

### Radiotherapy

Radiation was applied under general anaesthesia with intra-peritoneally administered 5% Pentobarbital sodium injection. A single dose of 18 Gy with 6 MV photon beams (Varian unique linear accelerator, USA) was applied via a single anterior field to 2 cm depth with source-axis distance technique based on the previous study^26^. 5 mm bolus was used to build-up the radiation dose on the lung. The radiation field included the right lung. The rats were sacrificed 4 weeks, 8 weeks and 16 weeks after radiation. The rats in the control group did not received radiation and sacrificed at the corresponding time point. The lung tissues were separated and stored at -80 °C for RNA sequencing.

### RNA sequencing

In order to explore the effects of radiotherapy in gene expression, rats’ total RNA was extracted and subjected to high-throughput sequencing based on Illumina HiSeq. 2500 platform to obtain RNA expression profile data. Total RNA was extracted from lung tissue samples using TRIzol reagent (Invitrogen) according to the manufacturer’s instruction as previously described^27^. The RNA was quantified using NanoDrop ND-1000 and assessed using a standard denaturing agarose gel electrophoresis assay. Then 1 μg of total RNA was used with the TruSeq RNA library preparation kit (Illumina) in accordance with low-throughput protocol, except that SuperScript III reverse transcriptase (Invitrogen) was used to synthesize first strand cDNA. After PCR enrichment and purification of adapter-ligated fragments, the concentration of DNA with adapters was determined with quantitative PCR (Applied Biosystems 7,500). Then, RNA sequencing was performed using the Illumina HiSeq. 2500 Sequencing System.

### Preprocessing and mapping of sequenced data

In order to ensure the data quality, raw reads were preprocessed by removing low quality sequences, de-junction contamination, rRNA removal, etc to obtain high quality sequences (clean reads) and all subsequent analyses were based on clean reads. Reference gene and genome annotation files were downloaded from the ENSEMBL website (http://www.ensembl.org/index.html). Clean Data was aligned to the reference genome by HISAT2 (http://ccb.jhu.edu/software/hisat2/index.shtml)^28^. HTSeq (http://www.huber.embl.de/users/anders/HTSeq/doc/overview.html) was used to estimate the expression level of each gene. The quantification of gene expression was performed using FPKM (Fragments Per Kilobase Millon Mapped Reads) method.

### Identification of differentially expressed genes

We used the DESseq. 2^29^ package in the R software to screen differentially expressed genes between radiotherapy group and controls. Data were normalized by a negative binomial distribution statistical method. The resulting P values were subjected to multiple test corrections according to the Benjamini and Hochberg methods to exclude false positives. The differentially expressed genes were identified when q ≤ 0.05 and |log2_ratio| ≥ 1.

### Screening for novel lncRNAs

We first identified the candidate lncRNAs using the following criterion: (1) reads with more than 200 bp and 2 exons; (2) transcripts with reads coverage ≤5 were excluded; (3) screening out known mRNAs and other non-coding RNAs (rRNA, tRNA, snoRNA, snRNA, etc.) in the samples using Gffcompare (http://ccb.jhu.edu/software/stringtie/gffcompare.shtml); (4) screening potential lincRNA, intronic lncRNA, anti-sense lncRNA according to the class code information (“u”, “i”, “x”) in the comparison results.

The coding ability was an important criterion for identifying lncRNA. Primary screening candidate lncRNAs were synthetically screened using the following softwares: CNCI analysis^30^, CPC^31^ analysis, PFAM^32^ protein domain analysis, CPAT^33^ analysis. The final novel lncRNA data set was composed of transcripts that were discriminated as non-coding by all four methods.

### Prediction for targets of lncRNA

The mRNAs located within 50 kb of the lncRNA were defined as potential cis targets and mRNAs with a correlation coefficient greater than 0.9 were defined as potential trans targets of lncRNA.

### Functional enrichment analysis of differentially expressed genes

Gene ontology (GO) analysis was applied to analyze the main function of differentially expressed genes. Based on the number of differentially expressed genes contained in each GO Term, a hypergeometric test was applied to find a significantly enriched GO Term compared to the entire genome background. The calculated P values were corrected by multiple tests. Functional terms with P < 0.05 were considered to be statistically significant.

KEGG (Kyoto Encyclopedia of Genes and Genomes, http://www.kegg.jp/) was a database of genome-wide metabolic pathways. Each pathway was analyzed by a hypergeometric test. The calculated P values were corrected by multiple tests. Pathways with P < 0.05 were considered to be statistically significant enriched.

### Tissue-specific analysis of differentially expressed genes

It was generally believed that the tissue specificity of lncRNAs was greater than protein-coding genes. Based on gene expression values, Jensen–Shannon divergence (JS score) of gene in tissues was calculated by a method of information entropy^34^. The maximum JS score of each gene was considered as its tissue-specific score. The tissue expression bias of genes was positively correlated with tissue-specific scores.

### Ethical approval

Animal experiments were granted an exemption from requiring ethics approval with National Cancer Center/National Clinical Research Center for Cancer/Cancer Hospital, Chinese Academy of Medical Science, Peking Union Medical College, the reference number is NCC2018A008.

## Results

### Sequencing reads quality control and mapping

**Figure 1:**
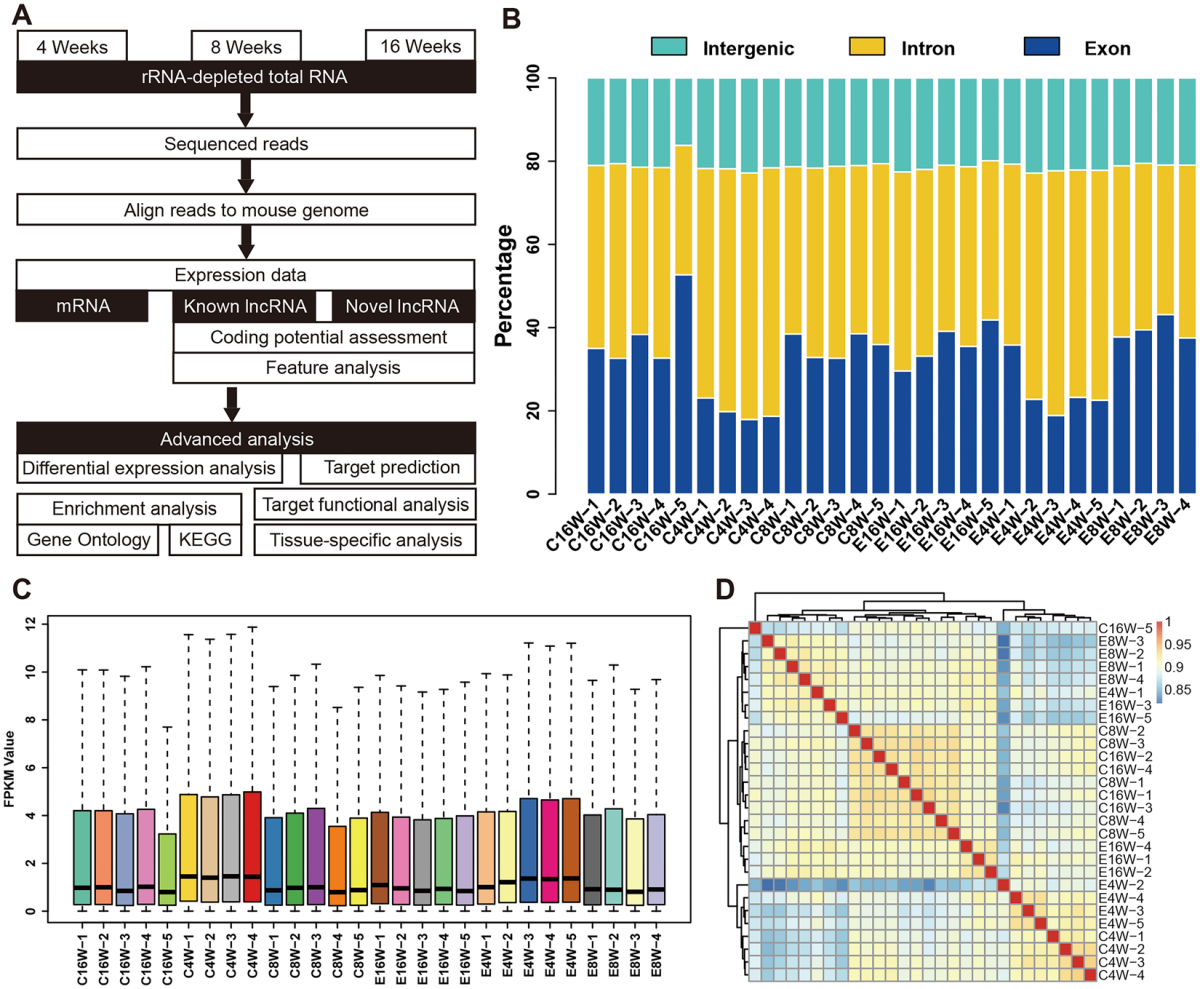
Data analysis process and quality control. (**A**) The rats were divided into three groups: radiotherapy for 4 weeks (nCon = 4, nexp = 5); radiotherapy for 8 weeks (nCon = 5, nexp = 4); radiotherapy for 16 weeks (nCon = 5, nexp = 5). Then rats’ lung tissues were processed with RNA extract, sequencing and function analysis; (**B**) The distribution of RNA-seq data in intron, exon and intergenic regions; (**C**) The distribution of gene expression values quantified by FPKM algorithm; (**D**) Heatmap of global mRNAs and lncRNAs in 28 clinical samples.

The basic sequencing and data analysis process was shown in Fig. 1A. The Raw reads were processed for quality control using Btrim to generate high-quality reads. Clean reads were mapped to reference genome and gene expression levels were evaluated. Fig. 1B showed that ∼35% (ranging from 17.93% to 52.71%) of total reads was distributed in the exon region. The remaining reads were mainly distributed in intron region (about 45%) and intergenic region (about 20%), which might be due to alternative splicing, expression noise and so on. The statistical analysis of clean reads indicated that the high-quality data ratio of all samples was above 90% (from 90.65% to 96.59%), except for one sample of C16W-5 (71.46%). Besides, the mapping rates of sequencing rates is high enough (95.11% to 96.43%) for the following analysis.

### Gene expression quantification

Gene expression level was characterized by FPKM (Fig. 1C). It was suggested that the expression levels of genes in all samples were overall consistent that suitable for differentially expressed analysis. The gene expression distribution was uniform. The present sequencing data were relatively effective for transcripts with expression levels between 1 and 5.

The similarity of gene expression between samples was also calculated, which indicated all control groups were highly similar, and samples in each experimental group were well clustered together (Fig. 1D). The heat map of whole genome RNA expression was shown in Fig. S1, which indicated that mRNA expression was relatively high and consistently distributed in each sample except for several individual mRNAs and lncRNA expression were relatively low and varied greatly among samples.

### Differentially expressed genes induced by radiation

**Figure 2:**
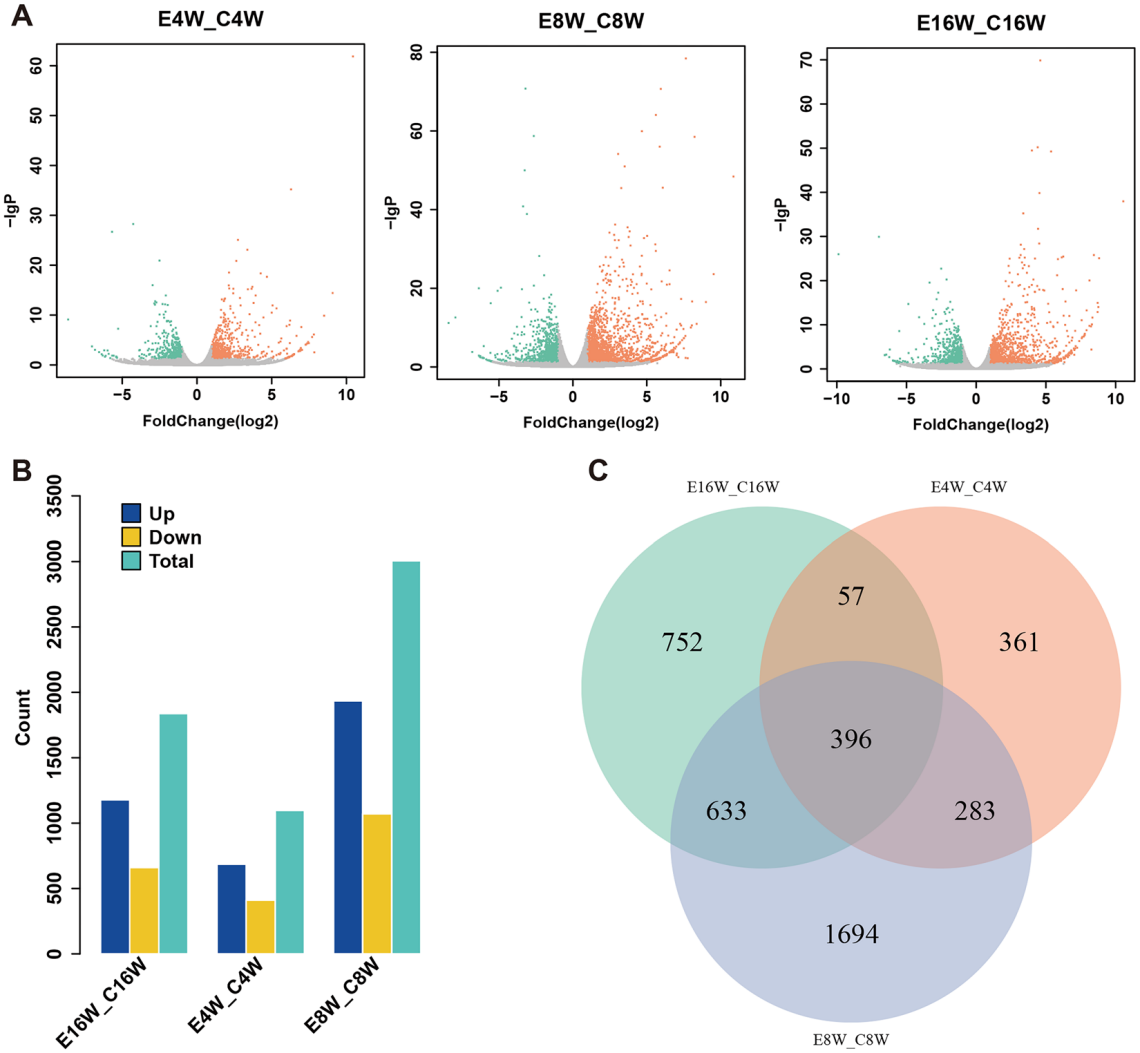
The analysis of differential expressed RNAs induced by radiotherapy. (**A**) The volcano map of differentially expressed genes in 4-week, 8-week and 16-week groups. X axis indicates the fold change of gene expression and Y axis indicates –lgP values. Red dots indicates high expression. Green dots indicates low expression. Gray dots indicates no significant change in expression; (**B**) The counts of differentially expressed genes in three groups; (**C**) The RNAs were identified from the intersection of the analysis results.

We further analyzed differentially expressed genes induced by radiation. From the heatmap in Fig. S1, one could intuitively observe a different expression pattern between radiation and control groups. Gene expression values between the radiation group and control group were compared using DESeq. 2. The distribution of differentially expressed genes was represented by a volcano map (Fig. 2A). Fig. 2B showed the total number of differentially expressed genes in three time points, which was 1097 (686 up- and 411 down-) for 4-week radiotherapy group, 3006 (1935 up- and 1071 down-) for 8-week group and 1838 (1178 up- and 660 down-) for 16-week group. Besides, differentially expressed genes had a certain intersection between different groups (Fig. 2C). The shared differentially expressed genes were 1029 between 4-week and 8-week group, 453 for 4-week and 16-week and 679 for 4-week and 8-week. A total of 396 differentially expressed genes were shared by three groups, which were listed in Table S1, including annotation and gene expression information.

**Figure 3:**
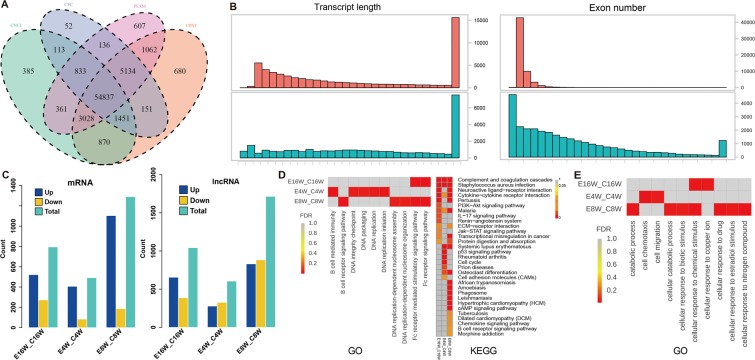
Function analysis of differentially expressed mRNAs and lncRNAs. (**A**) The novel lncRNAs were identified from the intersection of the analysis results by using CNCI, CPC, PFAM and CPAT; (**B**) The transcript length and exon number of differentially expressed lncRNAs and mRNAs; (**C**) The statistical results of differentially expressed lncRNAs and mRNAs; (**D**) GO (left) and KEGG (right) analysis of differentially expressed mRNAs; (**E**) The differentially expressed lncRNAs were performed target analysis and further GO term enrichment.

In order to obtain a more reliable novel lncRNA set, we used CNCI, CPC, PFAM, and CPAT to evaluate the coding potential of lncRNAs. The shared lncRNAs was used as the final novel lncRNA set, which finally resulted in a total of 54,837 lncRNAs (Fig. 3A). The transcript length and the number of exons in novel lncRNAs were shown in Fig. S2. The transcript length of novel lncRNA was mainly below 20 kb and exon number were distributed within 1-3, which was consistent with the typical characteristics of lncRNAs.

We further performed a structural analysis of lncRNAs and mRNAs (Fig. 3B bottom and upper panel, respectively). It was suggested that the transcript length and exon number of lncRNAs were significantly lower than those of mRNAs. The expression levels of mRNA and lncRNA in each sample was shown in Fig. S3, which indicated that the expression level of lncRNAs was significantly lower than that of mRNAs. Fig. 3C showed differentially expressed mRNAs and lncRNAs in different groups. There were 491 differentially expressed mRNAs (407 up- and 84 down-) and 606 lncRNAs (279 up- and 327 down-) in 4-week group. 1291 differentially expressed mRNAs (1104 up- and 187 down-) and 1715 lncRNAs (831 up- and 884 down-) were found in 8-week group. 795 differentially expressed mRNAs (522 up- and 273 down-) and 1043 lncRNAs (656 up- and 387 down-) were found in 16-week group.

To determine the primary functions of the differentially expressed genes, GO and KEGG pathway analysis was performed. As shown in Fig. 3D, signaling pathways were significantly enriched by the differentially expressed genes, including B cell mediated immunity, DNA integrity checkpoint, DNA packaging and DNA replication in 4-week group, B cell receptor signaling pathway, DNA replication related and Fc receptor related projects in 8-week group and Fc receptor mediated stimulatory signaling pathway, Fc receptor signaling pathway and other projects in 16-week group. For KEGG pathway, the differentially expressed genes of three groups were enriched in the metabolic pathways such as complement and coagulation cascades, Staphylococcus aureus infection, cytokine−cytokine receptor interaction and Malaria. A complete list of GO items for differentially expressed mRNA enrichment was shown in Fig. S4, mainly including three categories: biological processes, molecular functions, and cellular components.

LncRNA functions mainly through cis- or trans-acting on protein-coding genes. The statistical information of predicted target genes of lncRNAs was showed in Table S2. The functional enrichment analysis of cis- and trans- target of the differentially expressed lncRNAs were performed (Fig. 3E). The GO terms were mainly enriched in cell chemotaxis and cell migration pathways in 4-week group, catabolic process and cellular response related projects in 8-week group and cellular response related projects in 16-week group. For KEGG pathway, the target genes of lncRNAs were not significantly enriched. A complete list of GO items of target gene enrichment was shown in Fig. S5, which mainly included three categories: biological processes, molecular functions and cellular components. The predicted regulatory network among mRNA, lncRNA, and lncRNA target genes was shown in Fig. S6. The circles represented differentially expressed lncRNAs, and the squares represented their target genes and mRNAs. Upregulation was denoted by yellow, and downregulation was denoted by navy blue. It was indicated that the regulatory network of 8-week group was the most complex, while 4-week group presented a relatively simple regulatory network.

### Tissue specificity analysis

**Figure 4:**
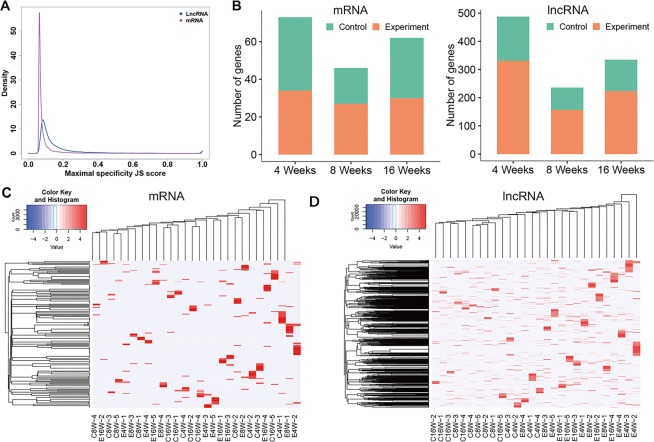
Tissue specificity analysis of differentially expressed mRNAs and lncRNAs. (**A**) Jensen–Shannon divergence (JS) distribution of differentially expressed mRNAs and lncRNAs; (**B**) The statistical analysis of differentially expressed mRNAs and lncRNAs with JS > 0.5; (**C**) Heatmap of differentially expressed mRNAs with JS > 0.5; (**D**) Heatmap of differentially expressed lncRNAs with JS > 0.

We next analyzed the tissue specificity of differentially expressed mRNAs and lncRNAs. The density distribution curve showed that lncRNAs had a significantly higher JS score than that of mRNAs (Fig. 4A), which was consistent with the strong tissue-specific expression of lncRNAs.

There were 73, 46, and 62 tissue-specific mRNAs (JS < 0.5) in 4-, 8- and 16-week groups, respectively (Fig. 4B). While the number of tissue-specific lncRNAs was respectively 488, 236 and 335 in 4-, 8- and 16-week groups, which indicated that lncRNAs had a higher tissue specificity than mRNAs. There were no significant differences in the number of tissue-specific mRNAs between the experimental and control groups in three groups. While, the number of tissue-specific lncRNAs in the experimental groups in the three groups was about twice that of the control group, suggesting that lncRNA might play an important role in radiotherapy. The information of tissue-specifically expressed mRNAs and lncRNAs were showed in Table S3 and Table S4.

The heat map in Fig. 4C showed the expression of tissue-specific mRNAs (JS > 0.5) in each sample and the expression of tissue-specific lncRNAs (JS > 0.5) was shown in Fig. 4D. The results showed that the tissue specificity of lncRNAs experimental groups was significantly higher than that in control groups, suggesting that lncRNA may play an important biological function in the experimental group.

## Discussion

Although radiotherapy effectively inhibits tumor growth in lung cancer patients, it also causes severe lung injury. To explore the molecular mechanism of radiotherapy for lung injury, we used RNA-seq to identify mRNA and lncRNA expression profile changes induced by radiation therapy in rats’ lung tissues. Comparing the transcriptome profiles in response to irradiation, we identified 491 (407 up- and 84 down-) differentially expressed mRNAs and 606 lncRNAs (279 up- and 327 down-) between the irradiated and control groups when radiotherapy for 4 weeks. 1291 differentially expressed mRNAs (1104 up- and 187 down-) and 1715 lncRNAs (831 up- and 884 down-) were found in 8-week group and 795 differentially expressed mRNAs (522 up- and 273 down-) and 1043 lncRNAs (656 up- and 387 down-) were found in 16-week group. There were 396 common differentially expressed genes (including mRNAs and lncRNAs) among the 4-week, 8-week and 16-week groups. Besides, compared with mRNAs, lncRNAs had shorter transcript length and less exon number, which was consistent with the general characteristics of lncRNAs. It is suggested that lncRNAs are genetic regulators of several biological processes, such as radiation-induced lung injury, and the differentially expressed lncRNAs are involved in this process by targeting related genes. For example, LIRR1, a differentially expressed lncRNA in radiation-induced lung injury, was associated with the altered expressions of KU70, KU80, RAD50, and MDM2^35^. Thus, the differentially expressed lncRNAs we screened might be significant biomarkers of radiation-induced lung injury.

Subsequently, the functions of the differentially expressed mRNAs and lncRNAs were analyzed. It was suggested that the differentially expressed mRNAs were implicated in several cellular processes including B cell mediated immunity, DNA integrity checkpoint, DNA packaging and DNA replication in 4-week group and Fc receptor signaling pathway in 8-week and 16-week groups. Cell cycle checkpoints play a critical role in orderly progression *via* regulating cell division^36,37^. These pathways respond to the adverse conditions by delaying or arresting cell cycles. It is indicated that the DNA damage checkpoint can arrest the cell cycle in order to suppress damaged DNA replication and chromosomes segregation that result in aneuploidy or instability of the genome^36–38^. DNA damage checkpoint consists of the following procedures: initiation, maintenance, and recovery, involving in multiple processes such as DNA lesion detection, signaling pathway activation, checkpoint signal maintenance, and checkpoint signal attenuation after repairment of DNA lesion. The procedures above are properly modulated to ensure the correct cooperation between cells and DNA damage events. Fc receptor, observed in various cells such as B lymphocytes and macrophages, is able to bind to the Fc region of antibodies and plays a protective role the immune system^39–41^. It is known that Fc receptor targets the antibodies that are attached to invading pathogens or infected cells, and induces destruction of microbes or infected cells *via* phagocytosis or cytotoxicity^27–29^. Therefore, we hypothesized that radiation therapy mainly contributed to arrestment of cell cycle and activation of the immune system in lung tissue. KEGG analysis results revealed that differentially expressed mRNAs were mainly involved in complement and coagulation cascades, staphylococcus aureus infection and cytokine−cytokine receptor interaction. GO analysis indicated that lncRNA target genes were associated with the regulation of cell migration and cellular stress response. However, for KEGG pathway, the lncRNAs target genes were not strikingly enriched. Furthermore, compared with control group, the tissue specificity of lncRNAs induced by radiation was significantly higher, suggesting that lncRNAs probably played a pivotal role in lung injury mechanism.

## Conclusions

A large amount of mRNAs and lncRNAs in the lung injury induced by radiation were identified in our study. Meanwhile, possible cell cycle regulation and immunological function for them were found during the pathogenesis of lung injury. Our results provided interesting clues on the mechanism of lung injury induced by radiation. Currently, the detailed effects of lncRNAs in radiation-induced lung injury have not been fully investigated yet, thus, our study might also provide promising information for future researches. As the current study aims to provide an overall analysis of the mRNAs and lncRNAs associated with early stage radiation-induced lung injury over time, our follow-up research would focus on several biomarkers from the significant mRNAs and lncRNAs screened to further investigate their specific roles in early stage of lung injury induced by radiation.

## Supplementary information

Supplementary Figures

Table S1

Table S2

Table S3

Table S4

## Data Availability

All data generated or analyzed during this study are included in this published article.
