# Supplementary material for: Transcriptome alteration spectrum in rat lung induced by radiotherapy
Source: Sci Rep. 2019 Dec 23;9:19701. doi: 10.1038/s41598-019-56027-4 (PMC6927959; doi:10.1038/s41598-019-56027-4)
Supplement: Supplementary file 1 — Supplementary Figures [file 41598_2019_56027_MOESM1_ESM.pdf]

# Transcriptome alteration spectrum in rat lung induced by radiotherapy

Tao Zhang[1], Guowei Cheng[2], Li Sun[2], Lei Deng[1], Xin Wang[1], Nan Bi[1]

1 Department of Radiation Oncology, National Cancer Center/National Clinical Research Center for Cancer/Cancer Hospital, Chinese Academy of Medical Science, Peking Union Medical College, Beijing, 100021, China.

2 Department of Radiation Oncology, Cancer Hospital of HuanXing ChaoYang District Beijing, Beijing, 100021, P.R. China.

Correspondence: Nan Bi

Department of Radiation Oncology, National Cancer Center/National Clinical Research Center for Cancer/Cancer Hospital, Chinese Academy of Medical Science, Peking Union Medical College, Beijing, 100021, China.

No.17 Panjiayuan Nanli, ChaoYang District, Beijing, 100021, China

E-mail: binan_email@163.com

Telephone number: （8610）87788995

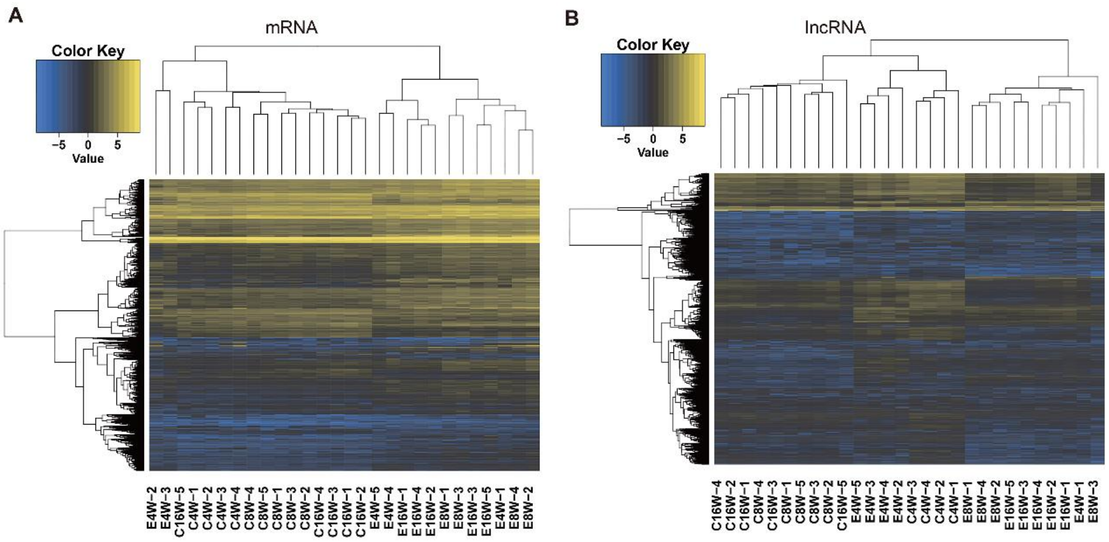


Figure S1 Heatmap of differentially expressed genes in all samples. (A) mRNAs; (B) lncRNAs.

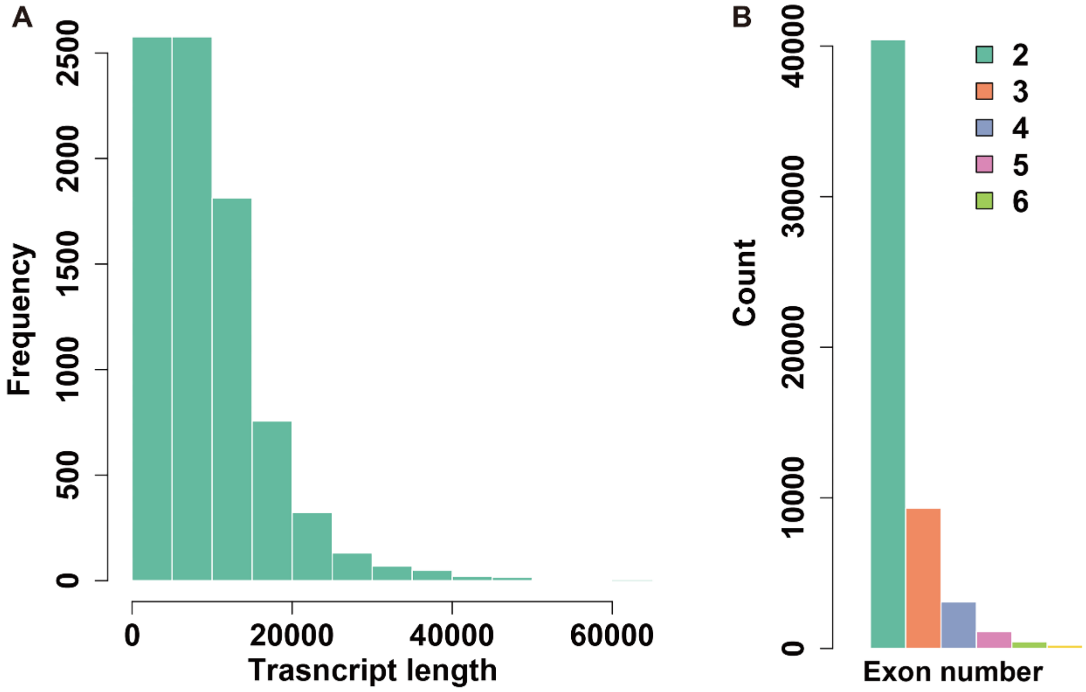


Figure S2 The length and exon number distribution of novel lncRNAs. (A) The frequencies of novel lncRNAs with different transcript length; (B) The counts of novel lncRNAs with different exon number.

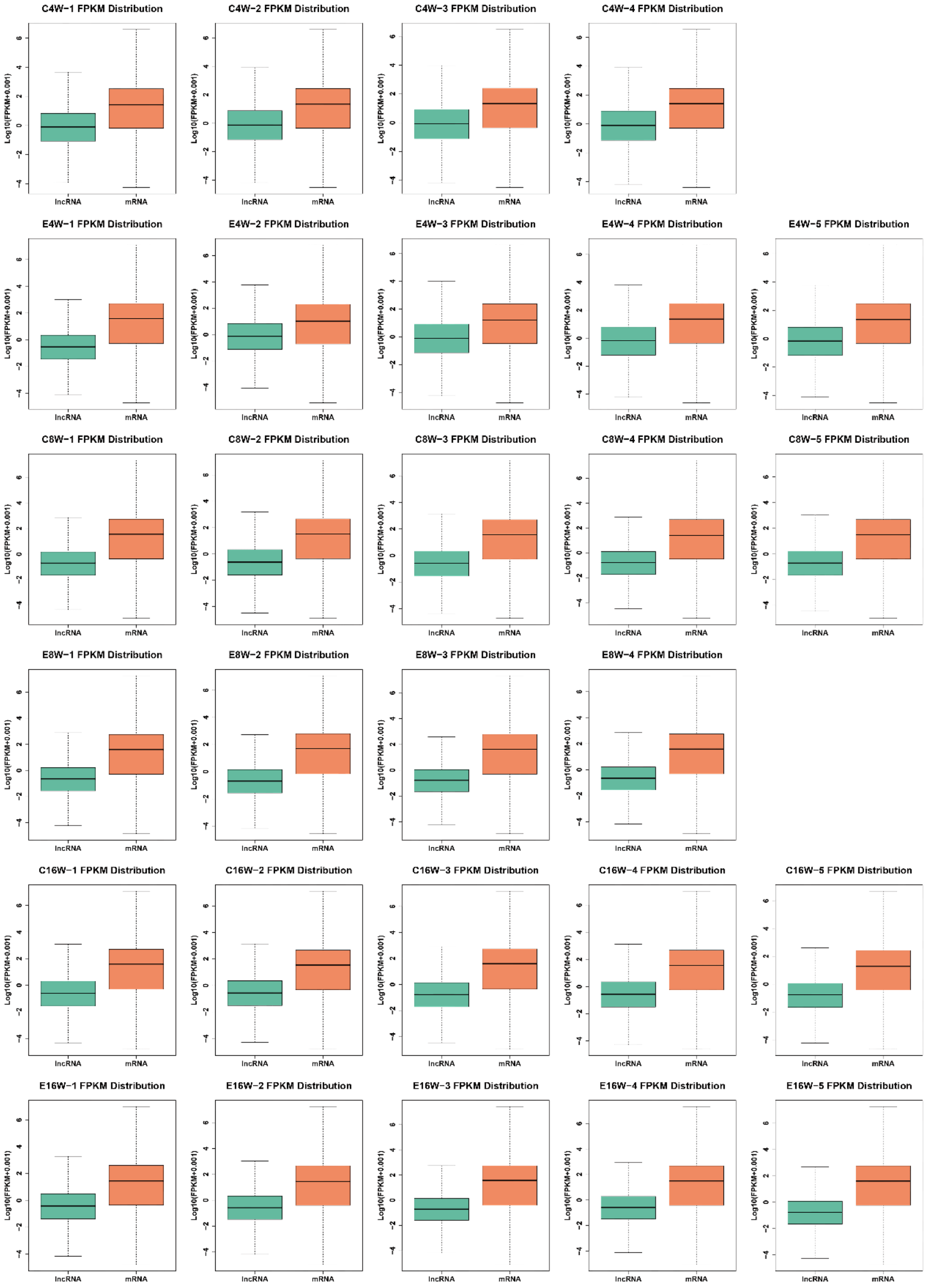


Figure S3 The comparison of gene expression value between mRNAs and lncRNAs in each sample.

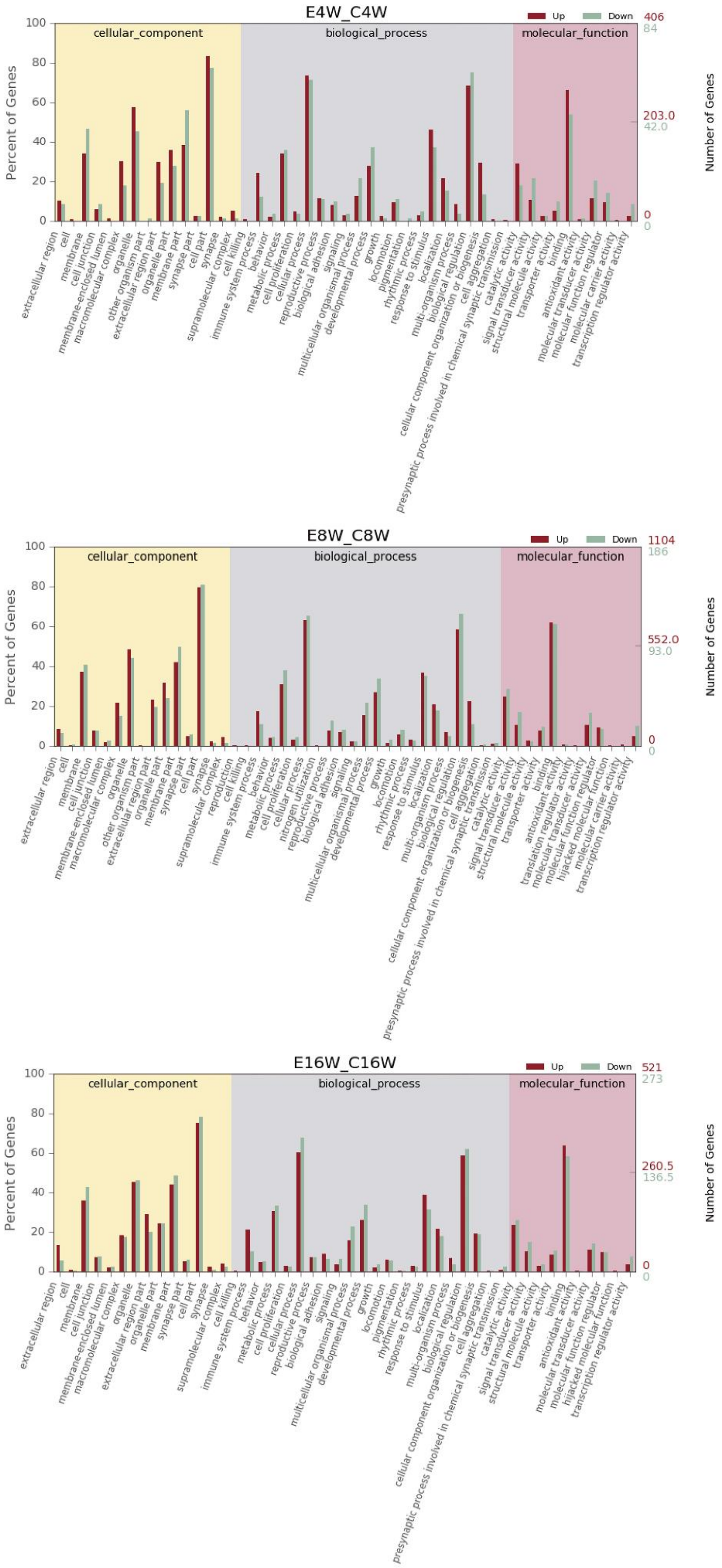


Figure S4 GO enrichment analysis for differentially expressed mRNAs.

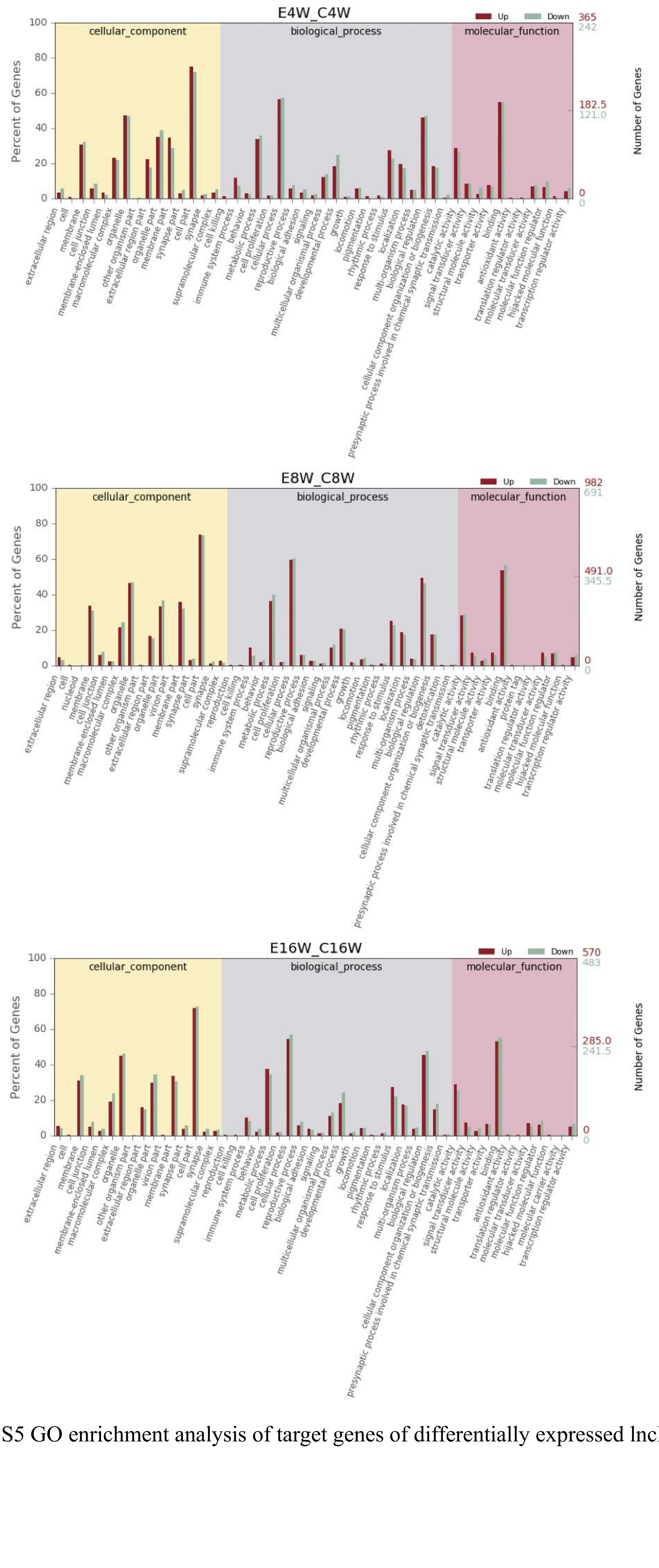


Figure S5 GO enrichment analysis of target genes of differentially expressed lncRNAs.

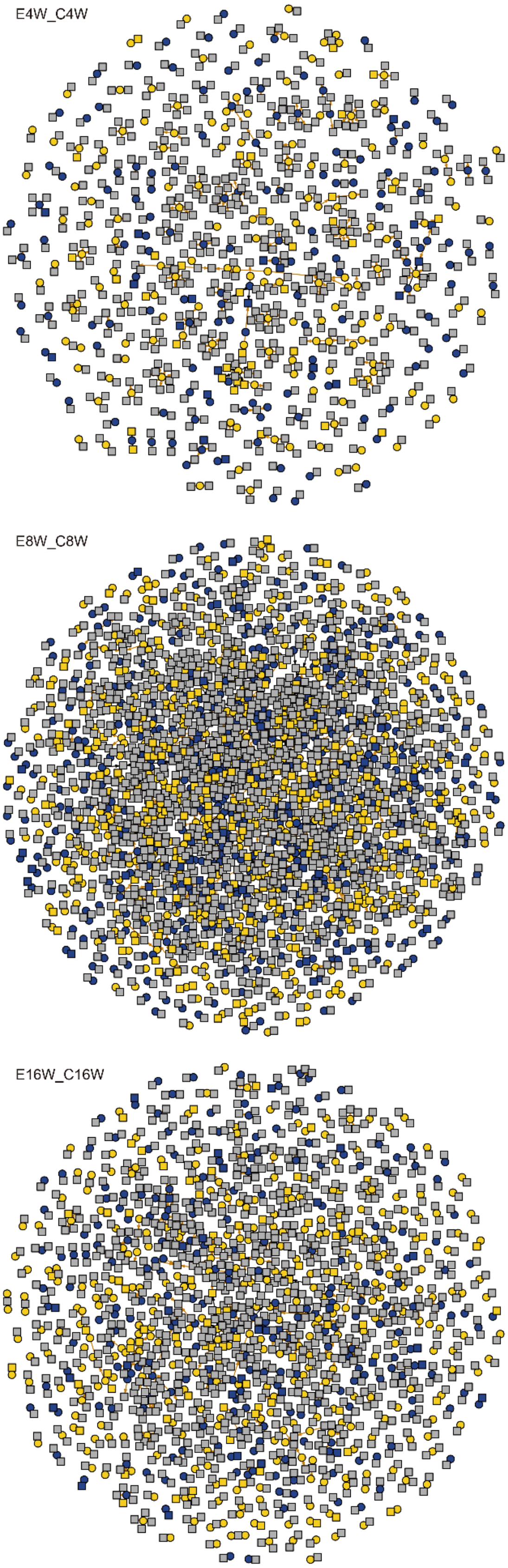


Figure S6 Differentially expressed mRNA, lncRNA, and The regulatory network of differentially expressed mRNAs and lncRNA cis and trans target genes.

**Figure legend**

Figure S1 Heatmap of differentially expressed genes in all samples. (A) mRNAs; (B) lncRNAs.

Figure S2 The length and exon number distribution of novel lncRNAs. (A) The frequencies of novel lncRNAs with different transcript length; (B) The counts of novel lncRNAs with different exon number.

Figure S3 The comparison of gene expression value between mRNAs and lncRNAs in each sample.

Figure S4 GO enrichment analysis for differentially expressed mRNAs.

Figure S5 GO enrichment analysis of target genes of differentially expressed lncRNAs.

Figure S6 Differentially expressed mRNA, lncRNA, and The regulatory network of differentially expressed mRNAs and lncRNA cis and trans target genes.
