# Supplementary material for: Transcriptome alteration spectrum in rat lung induced by radiotherapy
Source: Sci Rep. 2019 Dec 23;9:19701. doi: 10.1038/s41598-019-56027-4 (PMC6927959; doi:10.1038/s41598-019-56027-4)
Supplement: Supplementary file 2 — Table S1 [file 41598_2019_56027_MOESM2_ESM.pdf]

Transcriptome alteration spectrum in rat lung induced by radiotherapy

Tao Zhang[1], Guowei Cheng[2], Li Sun[2], Lei Deng[1], Xin Wang[1], Nan Bi[1]

1 Department of Radiation Oncology, National Cancer Center/National Clinical Research Center for Cancer/Cancer Hospital, Chinese Academy of Medical Science, Peking Union Medical College, Beijing, 100021, China.

2 Department of Radiation Oncology, Cancer Hospital of HuanXing ChaoYang District Beijing, Beijing, 100021, P.R. China.

Correspondence: Nan Bi

Department of Radiation Oncology, National Cancer Center/National Clinical Research Center for Cancer/Cancer Hospital, Chinese Academy of Medical Science, Peking Union Medical College, Beijing, 100021, China.

No.17 Panjiayuan Nanli, ChaoYang District, Beijing, 100021, China

E-mail: binan_email@163.com

Telephone number: （8610）87788995

Table S1 The differencially expressed genes shared by three groups.

Ensembl ID	Symbol	Ensembl ID	Symbol
ENSRNOG00000000187	Csf2rb	ENSRNOG00000018952	Sema3g
ENSRNOG00000000443	C4a	ENSRNOG00000019752	Slc29a1
ENSRNOG00000000521	Cdkn1a	ENSRNOG00000019890	Folr2
ENSRNOG00000001527	Cd80	ENSRNOG00000020552	Fosl1
ENSRNOG00000002434	Tmem100	ENSRNOG00000020563	Cma1
ENSRNOG00000002471	Polq	ENSRNOG00000020625	Mcpt2
ENSRNOG00000002653	Kcnk2	ENSRNOG00000020837	Cd300lg
ENSRNOG00000002711	Nuf2	ENSRNOG00000020991	Ms4a6a
ENSRNOG00000002771	Ereg	ENSRNOG00000020993	Ms4a2
ENSRNOG00000003135	Fcrlb	ENSRNOG00000021084	AABR07006310.1
ENSRNOG00000003229	Tspan7	ENSRNOG00000021155	Ctsk
ENSRNOG00000003251	B3galt2	ENSRNOG00000021243	Siglec1
ENSRNOG00000003365	Cadm3	ENSRNOG00000021569	Tiam1
ENSRNOG00000003388	Cenpf	ENSRNOG00000021663	RGD1561849
ENSRNOG00000003486	Mnda	ENSRNOG00000021669	Mybl1
ENSRNOG00000003666	Jchain	ENSRNOG00000021856	Lat2
ENSRNOG00000003872	Slc40a1	ENSRNOG00000021966	Il17rd
ENSRNOG00000003897	Col1a1	ENSRNOG00000021984	Rgs7
ENSRNOG00000003959	Rgs18	ENSRNOG00000022067	Tlr5
ENSRNOG00000004311	Gpr182	ENSRNOG00000022110	Gcsam
ENSRNOG00000004459	Sdr9c7	ENSRNOG00000022391	Zfp711
ENSRNOG00000004489	Adgre5	ENSRNOG00000022697	Clec14a
ENSRNOG00000004641	Sstr4	ENSRNOG00000022884	Cd84
ENSRNOG00000004695	Celf5	ENSRNOG00000023148	Col11a1
ENSRNOG00000004731	Ano3	ENSRNOG00000023712	Stox1
ENSRNOG00000004921	Nusap1	ENSRNOG00000024115	C6
ENSRNOG00000005277	Ptprv	ENSRNOG00000024181	Tpsab1
ENSRNOG00000005592	Brinp2	ENSRNOG00000024221	Mettl24
ENSRNOG00000005679	Fap	ENSRNOG00000024365	Ect2
ENSRNOG00000005723	Grin3a	ENSRNOG00000024428	Kif20a
ENSRNOG00000005780	Adamts13	ENSRNOG00000024650	Ckap2
ENSRNOG00000006583	Hpgds	ENSRNOG00000024785	Mcpt4
ENSRNOG00000006731	Spc25	ENSRNOG00000025130	Ltk
ENSRNOG00000006802	Lrrn1	ENSRNOG00000025198	Gas2l3
ENSRNOG00000007002	Lif	ENSRNOG00000025297	Gpr171
ENSRNOG00000007159	Ccl2	ENSRNOG00000025302	Cdca2
ENSRNOG00000007320	Fam3d	ENSRNOG00000026143	Ckap2l
ENSRNOG00000007637	Acer2	ENSRNOG00000026252	E2f7
ENSRNOG00000007765	Frzb	ENSRNOG00000026702	Jaml
ENSRNOG00000007906	Bub1b	ENSRNOG00000026953	Gpr88
ENSRNOG00000008045	Slamf9	ENSRNOG00000027035	Sgo2
ENSRNOG00000008165	Tpx2	ENSRNOG00000027098	Sez6l2
ENSRNOG00000008587	Tek	ENSRNOG00000027742	Adamtsl2
ENSRNOG00000008736	Slamf8	ENSRNOG00000028072	Chit1
ENSRNOG00000008890	Slc18a2	ENSRNOG00000028137	Mki67
ENSRNOG00000008933	Plbd1	ENSRNOG00000028616	Pck1
ENSRNOG00000008986	Diaph3	ENSRNOG00000029055	Ttk
ENSRNOG00000009008	Rab39a	ENSRNOG00000029212	Vcan
ENSRNOG00000009173	Smad6	ENSRNOG00000029756	P2ry13
ENSRNOG00000009177	Fcer1a	ENSRNOG00000030012	Clec4a2
ENSRNOG00000009227	Aplnr	ENSRNOG00000030187	Mmp12
ENSRNOG00000009334	Knstrn	ENSRNOG00000030387	Kng1
ENSRNOG00000009339	Cenpe	ENSRNOG00000030478	AY172581.9
ENSRNOG00000009503	Depdc1	ENSRNOG00000031304	Mcpt1l3
ENSRNOG00000010210	Slc7a11	ENSRNOG00000031443	Havcr2
ENSRNOG00000010253	Cd163	ENSRNOG00000031515	Klra2

ENSRNOG00000010262	Hdc	ENSRNOG00000031671	Rasgef1a
ENSRNOG00000010529	Thbs2	ENSRNOG00000032247	Bank1
ENSRNOG00000010797	Esm1	ENSRNOG00000032669	Serpina1
ENSRNOG00000010805	Fabp4	ENSRNOG00000033162	Chia
ENSRNOG00000011181	Cpa3	ENSRNOG00000033348	Duox1
ENSRNOG00000011381	Acsbg1	ENSRNOG00000033376	AABR07065782.1
ENSRNOG00000011478	Ackr4	ENSRNOG00000033658	Kntc1
ENSRNOG00000011672	Tph1	ENSRNOG00000037076	LOC689757
ENSRNOG00000011696	Lifr	ENSRNOG00000037211	Kif14
ENSRNOG00000011913	Cp	ENSRNOG00000037563	Cd68
ENSRNOG00000011946	Ptn	ENSRNOG00000038572	Ncapg
ENSRNOG00000012067	Fam111a	ENSRNOG00000039204	Pram1
ENSRNOG00000012109	Fam105a	ENSRNOG00000039740	Cenpk
ENSRNOG00000012749	C1qb	ENSRNOG00000039754	Rab7b
ENSRNOG00000012804	C1qc	ENSRNOG00000042628	RGD1561145
ENSRNOG00000013018	Eda2r	ENSRNOG00000042825	Cd300le
ENSRNOG00000013269	Tnfsf10	ENSRNOG00000042996	AABR07065798.1
ENSRNOG00000013399	LOC501033	ENSRNOG00000046452	Fcgr2b
ENSRNOG00000013413	Rorb	ENSRNOG00000046683	Lilrb3
ENSRNOG00000013641	Myo7a	ENSRNOG00000047457	Vipr1
ENSRNOG00000013684	F11	ENSRNOG00000047800	C5ar1
ENSRNOG00000013727	Ndc80	ENSRNOG00000048145	Sstr1
ENSRNOG00000013973	Lcn2	ENSRNOG00000048425	LOC691828
ENSRNOG00000014288	Fn1	ENSRNOG00000049063	Slc16a9
ENSRNOG00000014343	Anln	ENSRNOG00000049096	Mcpt8l2
ENSRNOG00000014648	Efnb2	ENSRNOG00000049115	Ccr5
ENSRNOG00000014653	Arl11	ENSRNOG00000049829	AABR07060872.1
ENSRNOG00000014835	Il1rl1	ENSRNOG00000049991	Mcpt8
ENSRNOG00000015308	Pbk	ENSRNOG00000050118	AABR07065750.2
ENSRNOG00000015411	Apobec1	ENSRNOG00000050708	AABR07065823.2
ENSRNOG00000015423	Ccna2	ENSRNOG00000052407	Btk
ENSRNOG00000016037	Mafb	ENSRNOG00000052887	Dnajc6
ENSRNOG00000016164	Fcrl2	ENSRNOG00000053026	Shcbp1
ENSRNOG00000016361	Plcd4	ENSRNOG00000053047	Top2a
ENSRNOG00000016377	Cep55	ENSRNOG00000053272	Chi3l1
ENSRNOG00000017485	Cd72	ENSRNOG00000053301	LOC685767
ENSRNOG00000017676	Plvap	ENSRNOG00000053494	Mcpt1l1
ENSRNOG00000018321	Sytl3	ENSRNOG00000054695	Calcrl
ENSRNOG00000018606	Olr59	ENSRNOG00000055512	Hist1h2bk
ENSRNOG00000018611	Tpsb2	ENSRNOG00000055796	AC103574.1
ENSRNOG00000018615	Cenph	ENSRNOG00000056069	Kif11
ENSRNOG00000018715	Clec10a	ENSRNOG00000056384	AABR07026612.1
ENSRNOG00000018929	Kif20b	ENSRNOG00000057376	AABR07051548.2

Ensembl ID	Symbol	Ensembl ID	Symbol
ENSRNOG00000058539	Ccnb1	MSTRG.281915	--
ENSRNOG00000058824	AABR07065789.3	MSTRG.283824	--
ENSRNOG00000059578	AC094636.1	MSTRG.289553	--
ENSRNOG00000059613	AABR07065780.1	MSTRG.295968	--
ENSRNOG00000060100	Knl1	MSTRG.301967	--
ENSRNOG00000061009	AABR07035790.2	MSTRG.307180	--
ENSRNOG00000062170	Pvt1	MSTRG.307398	--
MSTRG.107529	--	MSTRG.307400	--
MSTRG.108304	--	MSTRG.311008	--
MSTRG.108316	--	MSTRG.313145	--
MSTRG.112130	--	MSTRG.31355	--
MSTRG.113150	--	MSTRG.315189	--
MSTRG.115068	--	MSTRG.316121	--
MSTRG.118213	--	MSTRG.317271	--
MSTRG.120468	--	MSTRG.319903	--
MSTRG.120469	--	MSTRG.328015	--
MSTRG.120470	--	MSTRG.328016	--
MSTRG.121765	--	MSTRG.328017	--
MSTRG.123846	--	MSTRG.328803	--
MSTRG.133478	--	MSTRG.329396	--
MSTRG.133495	--	MSTRG.329400	--
MSTRG.133496	--	MSTRG.330094	--
MSTRG.133497	--	MSTRG.333925	--
MSTRG.137879	--	MSTRG.333926	--
MSTRG.138412	--	MSTRG.338411	--
MSTRG.138493	--	MSTRG.339503	--
MSTRG.142299	--	MSTRG.342138	--
MSTRG.143172	--	MSTRG.345468	--
MSTRG.143926	--	MSTRG.345521	--
MSTRG.143935	--	MSTRG.347346	--
MSTRG.143999	--	MSTRG.347366	--
MSTRG.144	--	MSTRG.347368	--
MSTRG.144023	--	MSTRG.347369	--
MSTRG.149188	--	MSTRG.347714	--
MSTRG.150947	--	MSTRG.348306	--
MSTRG.153735	--	MSTRG.351435	--
MSTRG.154614	--	MSTRG.351623	--
MSTRG.156491	--	MSTRG.351624	--
MSTRG.156501	--	MSTRG.352171	--
MSTRG.156502	--	MSTRG.360252	--
MSTRG.157323	--	MSTRG.360273	--
MSTRG.160029	--	MSTRG.361474	--
MSTRG.160447	--	MSTRG.362105	--
MSTRG.168985	--	MSTRG.364778	--
MSTRG.180710	--	MSTRG.365222	--
MSTRG.180816	--	MSTRG.365228	--
MSTRG.190424	--	MSTRG.365289	--
MSTRG.190430	--	MSTRG.372456	--
MSTRG.193580	--	MSTRG.375127	--
MSTRG.194105	--	MSTRG.375130	--
MSTRG.197851	--	MSTRG.375827	--
MSTRG.198313	--	MSTRG.38038	--
MSTRG.199226	--	MSTRG.38481	--
MSTRG.204338	--	MSTRG.389159	--
MSTRG.216034	--	MSTRG.389160	--
MSTRG.216038	--	MSTRG.389161	--

MSTRG.216039	--	MSTRG.390906	--
MSTRG.216041	--	MSTRG.396224	--
MSTRG.217408	--	MSTRG.398265	--
MSTRG.220503	--	MSTRG.398476	--
MSTRG.220684	--	MSTRG.404484	--
MSTRG.220685	--	MSTRG.40671	--
MSTRG.221008	--	MSTRG.45274	--
MSTRG.221464	--	MSTRG.49532	--
MSTRG.222219	--	MSTRG.54148	--
MSTRG.223595	--	MSTRG.54155	--
MSTRG.227707	--	MSTRG.54586	--
MSTRG.227713	--	MSTRG.57162	--
MSTRG.228412	--	MSTRG.57261	--
MSTRG.228413	--	MSTRG.67	--
MSTRG.230584	--	MSTRG.675	--
MSTRG.238335	--	MSTRG.67588	--
MSTRG.24139	--	MSTRG.69	--
MSTRG.24158	--	MSTRG.7046	--
MSTRG.243466	--	MSTRG.72436	--
MSTRG.247146	--	MSTRG.73379	--
MSTRG.247827	--	MSTRG.75665	--
MSTRG.247828	--	MSTRG.78029	--
MSTRG.252528	--	MSTRG.78315	--
MSTRG.25405	--	MSTRG.7881	--
MSTRG.254806	--	MSTRG.81449	--
MSTRG.25575	--	MSTRG.8526	--
MSTRG.261508	--	MSTRG.8529	--
MSTRG.261517	--	MSTRG.8548	--
MSTRG.261530	--	MSTRG.87641	--
MSTRG.26704	--	MSTRG.87644	--
MSTRG.26705	--	MSTRG.89056	--
MSTRG.26706	--	MSTRG.89323	--
MSTRG.26707	--	MSTRG.89330	--
MSTRG.26708	--	MSTRG.89422	--
MSTRG.267582	--	MSTRG.90242	--
MSTRG.26927	--	MSTRG.9657	--
MSTRG.275659	--	MSTRG.97398	--
MSTRG.276478	--	MSTRG.97399	--
MSTRG.276615	--	MSTRG.97401	--
MSTRG.276631	--	MSTRG.97409	--
MSTRG.280435	--	MSTRG.97765	--
MSTRG.28047	--	MSTRG.97820	--
MSTRG.280575	--	MSTRG.9958	--
