# Supplementary material for: Transcriptome alteration spectrum in rat lung induced by radiotherapy
Source: Sci Rep. 2019 Dec 23;9:19701. doi: 10.1038/s41598-019-56027-4 (PMC6927959; doi:10.1038/s41598-019-56027-4)
Supplement: Supplementary file 3 — Table S2 [file 41598_2019_56027_MOESM3_ESM.pdf]

Transcriptome alteration spectrum in rat lung induced by radiotherapy

Tao Zhang[1], Guowei Cheng[2], Li Sun[2], Lei Deng[1], Xin Wang[1], Nan Bi[1]

1 Department of Radiation Oncology, National Cancer Center/National Clinical Research Center for Cancer/Cancer Hospital, Chinese Academy of Medical Science, Peking Union Medical College, Beijing, 100021, China.

2 Department of Radiation Oncology, Cancer Hospital of HuanXing ChaoYang District Beijing, Beijing, 100021, P.R. China.

Correspondence: Nan Bi

Department of Radiation Oncology, National Cancer Center/National Clinical Research Center for Cancer/Cancer Hospital, Chinese Academy of Medical Science, Peking Union Medical College, Beijing, 100021, China.

No.17 Panjiayuan Nanli, ChaoYang District, Beijing, 100021, China

E-mail: binan_email@163.com

Telephone number: （8610）87788995

Table S2 Distribution of cis- and trans-targets of DElncRNA

Compare	Total DE	LncRNA D	Cistarget	Transtarget
E16W_C16	1838	1043	1064	31
E4W_C4W	1097	606	611	26
E8W_C8W	3006	1715	1622	99
