# Supplementary material for: Transcriptome alteration spectrum in rat lung induced by radiotherapy
Source: Sci Rep. 2019 Dec 23;9:19701. doi: 10.1038/s41598-019-56027-4 (PMC6927959; doi:10.1038/s41598-019-56027-4)
Supplement: Supplementary file 4 — Table S3 [file 41598_2019_56027_MOESM4_ESM.pdf]

Transcriptome alteration spectrum in rat lung induced by radiotherapy

Tao Zhang[1], Guowei Cheng[2], Li Sun[2], Lei Deng[1], Xin Wang[1], Nan Bi[1]

1 Department of Radiation Oncology, National Cancer Center/National Clinical Research Center for Cancer/Cancer Hospital, Chinese Academy of Medical Science, Peking Union Medical College, Beijing, 100021, China.

2 Department of Radiation Oncology, Cancer Hospital of HuanXing ChaoYang District Beijing, Beijing, 100021, P.R. China.

Correspondence: Nan Bi

Department of Radiation Oncology, National Cancer Center/National Clinical Research Center for Cancer/Cancer Hospital, Chinese Academy of Medical Science, Peking Union Medical College, Beijing, 100021, China.

No.17 Panjiayuan Nanli, ChaoYang District, Beijing, 100021, China

E-mail: binan_email@163.com

Telephone number: （8610）87788995

Table S3 Tissue-specifically expressed mRNAs.

GeneID	GeneID	GeneID
ENSRNOG00000009160	ENSRNOG00000038376	ENSRNOG00000047809
ENSRNOG00000019037	ENSRNOG00000040355	ENSRNOG00000047886
ENSRNOG00000023090	ENSRNOG00000040387	ENSRNOG00000048030
ENSRNOG00000029389	ENSRNOG00000041292	ENSRNOG00000048183
ENSRNOG00000032835	ENSRNOG00000041450	ENSRNOG00000048240
ENSRNOG00000033938	ENSRNOG00000041523	ENSRNOG00000048306
ENSRNOG00000034695	ENSRNOG00000041549	ENSRNOG00000048338
ENSRNOG00000035458	ENSRNOG00000043109	ENSRNOG00000048484
ENSRNOG00000035485	ENSRNOG00000043707	ENSRNOG00000048568
ENSRNOG00000035487	ENSRNOG00000044833	ENSRNOG00000048587
ENSRNOG00000035510	ENSRNOG00000044850	ENSRNOG00000048618
ENSRNOG00000035530	ENSRNOG00000045356	ENSRNOG00000049185
ENSRNOG00000035557	ENSRNOG00000045581	ENSRNOG00000049253
ENSRNOG00000035570	ENSRNOG00000045701	ENSRNOG00000049341
ENSRNOG00000035648	ENSRNOG00000045749	ENSRNOG00000049542
ENSRNOG00000036328	ENSRNOG00000045809	ENSRNOG00000049638
ENSRNOG00000036404	ENSRNOG00000046071	ENSRNOG00000049844
ENSRNOG00000036483	ENSRNOG00000046121	ENSRNOG00000050541
ENSRNOG00000036489	ENSRNOG00000047351	ENSRNOG00000050734
ENSRNOG00000036530	ENSRNOG00000047435	ENSRNOG00000050873

GeneID	GeneID	GeneID
ENSRNOG00000051034	ENSRNOG00000053461	ENSRNOG00000055708
ENSRNOG00000051493	ENSRNOG00000053484	ENSRNOG00000055727
ENSRNOG00000051538	ENSRNOG00000053555	ENSRNOG00000055817
ENSRNOG00000051641	ENSRNOG00000053573	ENSRNOG00000055956
ENSRNOG00000051649	ENSRNOG00000054300	ENSRNOG00000055979
ENSRNOG00000051781	ENSRNOG00000054310	ENSRNOG00000056002
ENSRNOG00000051811	ENSRNOG00000054326	ENSRNOG00000056046
ENSRNOG00000051864	ENSRNOG00000054816	ENSRNOG00000056068
ENSRNOG00000051930	ENSRNOG00000054892	ENSRNOG00000056100
ENSRNOG00000052007	ENSRNOG00000054982	ENSRNOG00000056160
ENSRNOG00000052097	ENSRNOG00000055100	ENSRNOG00000056178
ENSRNOG00000052172	ENSRNOG00000055183	ENSRNOG00000056202
ENSRNOG00000052185	ENSRNOG00000055276	ENSRNOG00000056308
ENSRNOG00000052320	ENSRNOG00000055311	ENSRNOG00000056405
ENSRNOG00000052808	ENSRNOG00000055354	ENSRNOG00000056412
ENSRNOG00000052956	ENSRNOG00000055360	ENSRNOG00000056426
ENSRNOG00000052983	ENSRNOG00000055424	ENSRNOG00000056437
ENSRNOG00000053153	ENSRNOG00000055558	ENSRNOG00000056499
ENSRNOG00000053319	ENSRNOG00000055626	ENSRNOG00000056664
ENSRNOG00000053408	ENSRNOG00000055667	ENSRNOG00000056684

GeneID	GeneID	GeneID
ENSRNOG00000056695	ENSRNOG00000057912	ENSRNOG00000060170
ENSRNOG00000056719	ENSRNOG00000058041	ENSRNOG00000060177
ENSRNOG00000056737	ENSRNOG00000058349	ENSRNOG00000060516
ENSRNOG00000056760	ENSRNOG00000058536	ENSRNOG00000060600
ENSRNOG00000056890	ENSRNOG00000058657	ENSRNOG00000060621
ENSRNOG00000056912	ENSRNOG00000058756	ENSRNOG00000060659
ENSRNOG00000057225	ENSRNOG00000058757	ENSRNOG00000060660
ENSRNOG00000057248	ENSRNOG00000058858	ENSRNOG00000061023
ENSRNOG00000057331	ENSRNOG00000058888	ENSRNOG00000061156
ENSRNOG00000057336	ENSRNOG00000059012	ENSRNOG00000061202
ENSRNOG00000057354	ENSRNOG00000059108	ENSRNOG00000061385
ENSRNOG00000057414	ENSRNOG00000059256	ENSRNOG00000061471
ENSRNOG00000057516	ENSRNOG00000059287	ENSRNOG00000061478
ENSRNOG00000057559	ENSRNOG00000059394	ENSRNOG00000061482
ENSRNOG00000057567	ENSRNOG00000059451	ENSRNOG00000061748
ENSRNOG00000057571	ENSRNOG00000059488	ENSRNOG00000061852
ENSRNOG00000057735	ENSRNOG00000059521	ENSRNOG00000061864
ENSRNOG00000057743	ENSRNOG00000059819	ENSRNOG00000061931
ENSRNOG00000057808	ENSRNOG00000059990	
ENSRNOG00000057819	ENSRNOG00000060094	
