# Supplementary material for: Transcriptome alteration spectrum in rat lung induced by radiotherapy
Source: Sci Rep. 2019 Dec 23;9:19701. doi: 10.1038/s41598-019-56027-4 (PMC6927959; doi:10.1038/s41598-019-56027-4)
Supplement: Supplementary file 5 — Table S4 [file 41598_2019_56027_MOESM5_ESM.pdf]

Transcriptome alteration spectrum in rat lung induced by radiotherapy

Tao Zhang[1], Guowei Cheng[2], Li Sun[2], Lei Deng[1], Xin Wang[1], Nan Bi[1]

1 Department of Radiation Oncology, National Cancer Center/National Clinical Research Center for Cancer/Cancer Hospital, Chinese Academy of Medical Science, Peking Union Medical College, Beijing, 100021, China.

2 Department of Radiation Oncology, Cancer Hospital of HuanXing ChaoYang District Beijing, Beijing, 100021, P.R. China.

Correspondence: Nan Bi

Department of Radiation Oncology, National Cancer Center/National Clinical Research Center for Cancer/Cancer Hospital, Chinese Academy of Medical Science, Peking Union Medical College, Beijing, 100021, China.

No.17 Panjiayuan Nanli, ChaoYang District, Beijing, 100021, China

E-mail: binan_email@163.com

Telephone number: （8610）87788995

Table S4 Tissue-specifically expressed lncRNAs.

GeneID	GeneID	GeneID	GeneID	GeneID
ENSRNOG00000051397	MSTRG.136110	MSTRG.177476	MSTRG.207073	MSTRG.24881
ENSRNOG00000052323	MSTRG.13670	MSTRG.177970	MSTRG.207083	MSTRG.249326
ENSRNOG00000052452	MSTRG.137329	MSTRG.178159	MSTRG.207613	MSTRG.249540
ENSRNOG00000052630	MSTRG.137484	MSTRG.1782	MSTRG.208590	MSTRG.249981
ENSRNOG00000052693	MSTRG.137659	MSTRG.17860	MSTRG.20899	MSTRG.250979
ENSRNOG00000052901	MSTRG.137722	MSTRG.178870	MSTRG.209198	MSTRG.251434
ENSRNOG00000052940	MSTRG.137881	MSTRG.179259	MSTRG.209555	MSTRG.25194
ENSRNOG00000054396	MSTRG.137963	MSTRG.180094	MSTRG.209752	MSTRG.252031
ENSRNOG00000054658	MSTRG.138375	MSTRG.180293	MSTRG.209937	MSTRG.252354
ENSRNOG00000055842	MSTRG.138574	MSTRG.180372	MSTRG.209974	MSTRG.25243
ENSRNOG00000056522	MSTRG.138649	MSTRG.180740	MSTRG.21028	MSTRG.252501
ENSRNOG00000057876	MSTRG.138742	MSTRG.180814	MSTRG.21046	MSTRG.252917
ENSRNOG00000058557	MSTRG.138769	MSTRG.180913	MSTRG.210608	MSTRG.252968
ENSRNOG00000060165	MSTRG.138822	MSTRG.181065	MSTRG.210806	MSTRG.253441
ENSRNOG00000061542	MSTRG.139242	MSTRG.181240	MSTRG.210821	MSTRG.253584
MSTRG.100823	MSTRG.139448	MSTRG.182052	MSTRG.211353	MSTRG.253597
MSTRG.101463	MSTRG.139508	MSTRG.182087	MSTRG.21146	MSTRG.253736
MSTRG.101593	MSTRG.139622	MSTRG.182250	MSTRG.211786	MSTRG.253998
MSTRG.101808	MSTRG.140044	MSTRG.182869	MSTRG.212044	MSTRG.254595
MSTRG.101919	MSTRG.14099	MSTRG.183022	MSTRG.21209	MSTRG.254653
MSTRG.102040	MSTRG.141046	MSTRG.183211	MSTRG.212650	MSTRG.255810
MSTRG.102366	MSTRG.141110	MSTRG.183795	MSTRG.212882	MSTRG.255973
MSTRG.1035	MSTRG.141385	MSTRG.183971	MSTRG.213456	MSTRG.256128
MSTRG.104157	MSTRG.141574	MSTRG.184480	MSTRG.21382	MSTRG.256189
MSTRG.104282	MSTRG.141639	MSTRG.184779	MSTRG.213820	MSTRG.256635
MSTRG.104694	MSTRG.142290	MSTRG.185178	MSTRG.214554	MSTRG.25673
MSTRG.105660	MSTRG.144598	MSTRG.185572	MSTRG.215085	MSTRG.256856
MSTRG.105757	MSTRG.145536	MSTRG.185680	MSTRG.215205	MSTRG.256998
MSTRG.105938	MSTRG.147302	MSTRG.185801	MSTRG.215754	MSTRG.259000
MSTRG.106010	MSTRG.147983	MSTRG.186188	MSTRG.215987	MSTRG.260541
MSTRG.106778	MSTRG.148208	MSTRG.186257	MSTRG.21622	MSTRG.261051
MSTRG.107062	MSTRG.148260	MSTRG.186496	MSTRG.216390	MSTRG.261243
MSTRG.107109	MSTRG.148673	MSTRG.187068	MSTRG.216573	MSTRG.262067
MSTRG.107128	MSTRG.148896	MSTRG.187415	MSTRG.217082	MSTRG.262386
MSTRG.108284	MSTRG.149067	MSTRG.187762	MSTRG.217083	MSTRG.262414
MSTRG.108493	MSTRG.149132	MSTRG.187848	MSTRG.217573	MSTRG.262620
MSTRG.108545	MSTRG.149325	MSTRG.188372	MSTRG.218169	MSTRG.262971
MSTRG.108691	MSTRG.149379	MSTRG.188385	MSTRG.218541	MSTRG.263167
MSTRG.108983	MSTRG.150781	MSTRG.188720	MSTRG.218694	MSTRG.263190
MSTRG.10985	MSTRG.151335	MSTRG.188790	MSTRG.219042	MSTRG.263249
MSTRG.110224	MSTRG.151699	MSTRG.189157	MSTRG.220159	MSTRG.263520
MSTRG.112037	MSTRG.151887	MSTRG.189429	MSTRG.220426	MSTRG.26465
MSTRG.112853	MSTRG.152042	MSTRG.190455	MSTRG.220933	MSTRG.264793
MSTRG.113399	MSTRG.152344	MSTRG.190890	MSTRG.221187	MSTRG.264857
MSTRG.113538	MSTRG.152899	MSTRG.19095	MSTRG.221637	MSTRG.265782
MSTRG.113747	MSTRG.153176	MSTRG.191169	MSTRG.221909	MSTRG.266123
MSTRG.113968	MSTRG.153296	MSTRG.191361	MSTRG.22203	MSTRG.266133
MSTRG.114188	MSTRG.153440	MSTRG.191642	MSTRG.222263	MSTRG.267707
MSTRG.114508	MSTRG.154430	MSTRG.191742	MSTRG.222466	MSTRG.267964
MSTRG.11464	MSTRG.154818	MSTRG.191803	MSTRG.222784	MSTRG.26841
MSTRG.114669	MSTRG.155560	MSTRG.192121	MSTRG.223070	MSTRG.268817
MSTRG.115273	MSTRG.156008	MSTRG.192163	MSTRG.224252	MSTRG.269827
MSTRG.117205	MSTRG.156063	MSTRG.192937	MSTRG.224466	MSTRG.26984
MSTRG.117280	MSTRG.156331	MSTRG.193611	MSTRG.224511	MSTRG.269868
MSTRG.117717	MSTRG.15702	MSTRG.193743	MSTRG.224533	MSTRG.270007
MSTRG.117763	MSTRG.157048	MSTRG.194156	MSTRG.224623	MSTRG.270481

MSTRG.118050	MSTRG.157305	MSTRG.194284	MSTRG.225060	MSTRG.270724
MSTRG.118461	MSTRG.158158	MSTRG.194473	MSTRG.225477	MSTRG.270841
MSTRG.119066	MSTRG.159066	MSTRG.194540	MSTRG.225929	MSTRG.271711
MSTRG.119582	MSTRG.159357	MSTRG.194549	MSTRG.226053	MSTRG.272306
MSTRG.119800	MSTRG.159749	MSTRG.194775	MSTRG.22648	MSTRG.272527
MSTRG.119822	MSTRG.160017	MSTRG.195036	MSTRG.226677	MSTRG.272702
MSTRG.120225	MSTRG.160129	MSTRG.195472	MSTRG.226921	MSTRG.273439
MSTRG.120392	MSTRG.160136	MSTRG.195585	MSTRG.227650	MSTRG.273554
MSTRG.121314	MSTRG.160444	MSTRG.196673	MSTRG.227930	MSTRG.273710
MSTRG.121413	MSTRG.160707	MSTRG.197074	MSTRG.22861	MSTRG.27376
MSTRG.121730	MSTRG.160846	MSTRG.197924	MSTRG.228709	MSTRG.273762
MSTRG.121804	MSTRG.161308	MSTRG.198077	MSTRG.229330	MSTRG.274431
MSTRG.122164	MSTRG.161360	MSTRG.198315	MSTRG.229483	MSTRG.274754
MSTRG.122699	MSTRG.161700	MSTRG.198645	MSTRG.230263	MSTRG.275149
MSTRG.122702	MSTRG.16221	MSTRG.198711	MSTRG.230492	MSTRG.275434
MSTRG.12272	MSTRG.162233	MSTRG.198964	MSTRG.231640	MSTRG.275908
MSTRG.122720	MSTRG.162744	MSTRG.199101	MSTRG.231991	MSTRG.276149
MSTRG.122811	MSTRG.162777	MSTRG.199745	MSTRG.232892	MSTRG.27625
MSTRG.122834	MSTRG.164038	MSTRG.200069	MSTRG.233032	MSTRG.277163
MSTRG.124053	MSTRG.164452	MSTRG.200432	MSTRG.234107	MSTRG.277447
MSTRG.124527	MSTRG.165567	MSTRG.20067	MSTRG.235719	MSTRG.277761
MSTRG.124830	MSTRG.166309	MSTRG.2008	MSTRG.236017	MSTRG.277774
MSTRG.125276	MSTRG.166321	MSTRG.200812	MSTRG.236295	MSTRG.277956
MSTRG.125596	MSTRG.167421	MSTRG.200865	MSTRG.236450	MSTRG.278173
MSTRG.125857	MSTRG.167825	MSTRG.200940	MSTRG.236998	MSTRG.278196
MSTRG.125952	MSTRG.168105	MSTRG.201061	MSTRG.237336	MSTRG.278759
MSTRG.126694	MSTRG.168797	MSTRG.201194	MSTRG.237871	MSTRG.279321
MSTRG.126759	MSTRG.169110	MSTRG.201347	MSTRG.237880	MSTRG.279694
MSTRG.12696	MSTRG.169385	MSTRG.201370	MSTRG.239166	MSTRG.280156
MSTRG.127807	MSTRG.169388	MSTRG.201493	MSTRG.24016	MSTRG.28040
MSTRG.128227	MSTRG.169882	MSTRG.201785	MSTRG.240425	MSTRG.28067
MSTRG.128550	MSTRG.170001	MSTRG.201925	MSTRG.241493	MSTRG.280676
MSTRG.128958	MSTRG.170002	MSTRG.202645	MSTRG.242647	MSTRG.280886
MSTRG.129039	MSTRG.170320	MSTRG.20265	MSTRG.242651	MSTRG.2810
MSTRG.129482	MSTRG.171382	MSTRG.202653	MSTRG.242682	MSTRG.281370
MSTRG.129570	MSTRG.171417	MSTRG.202720	MSTRG.24371	MSTRG.281449
MSTRG.130149	MSTRG.172012	MSTRG.203566	MSTRG.244389	MSTRG.281609
MSTRG.130280	MSTRG.172706	MSTRG.203614	MSTRG.244610	MSTRG.282844
MSTRG.130311	MSTRG.174167	MSTRG.204167	MSTRG.245034	MSTRG.282867
MSTRG.130348	MSTRG.17555	MSTRG.204677	MSTRG.245905	MSTRG.282980
MSTRG.130539	MSTRG.17568	MSTRG.204723	MSTRG.246010	MSTRG.284699
MSTRG.130782	MSTRG.17605	MSTRG.204857	MSTRG.246139	MSTRG.284728
MSTRG.131638	MSTRG.176234	MSTRG.205207	MSTRG.246187	MSTRG.285175
MSTRG.132000	MSTRG.176346	MSTRG.205962	MSTRG.246293	MSTRG.285602
MSTRG.1322	MSTRG.176692	MSTRG.206374	MSTRG.246438	MSTRG.285713
MSTRG.132665	MSTRG.176710	MSTRG.206446	MSTRG.247391	MSTRG.286503
MSTRG.135166	MSTRG.176936	MSTRG.2066	MSTRG.248629	MSTRG.286713
MSTRG.135624	MSTRG.177028	MSTRG.206784	MSTRG.248701	MSTRG.286886
MSTRG.135714	MSTRG.177205	MSTRG.206883	MSTRG.248734	MSTRG.286993

GeneID	GeneID	GeneID	GeneID	GeneID
MSTRG.287230	MSTRG.318856	MSTRG.354381	MSTRG.391400	MSTRG.66021
MSTRG.28762	MSTRG.319917	MSTRG.35451	MSTRG.391869	MSTRG.66095
MSTRG.287888	MSTRG.320795	MSTRG.354763	MSTRG.394648	MSTRG.66143
MSTRG.288185	MSTRG.320853	MSTRG.355707	MSTRG.39520	MSTRG.66172
MSTRG.289447	MSTRG.320989	MSTRG.355975	MSTRG.396903	MSTRG.66378
MSTRG.28948	MSTRG.321270	MSTRG.356388	MSTRG.397192	MSTRG.66677
MSTRG.289889	MSTRG.321316	MSTRG.356429	MSTRG.397282	MSTRG.67483
MSTRG.290422	MSTRG.321633	MSTRG.356538	MSTRG.39754	MSTRG.67602
MSTRG.29046	MSTRG.322154	MSTRG.356674	MSTRG.398042	MSTRG.67759
MSTRG.290809	MSTRG.322353	MSTRG.357589	MSTRG.398184	MSTRG.68142
MSTRG.291394	MSTRG.323764	MSTRG.357636	MSTRG.399381	MSTRG.68169
MSTRG.291643	MSTRG.323932	MSTRG.357774	MSTRG.399927	MSTRG.6872
MSTRG.291721	MSTRG.323965	MSTRG.357943	MSTRG.400025	MSTRG.68828
MSTRG.292438	MSTRG.324104	MSTRG.357970	MSTRG.401541	MSTRG.68877
MSTRG.292652	MSTRG.324137	MSTRG.358609	MSTRG.401680	MSTRG.691
MSTRG.292653	MSTRG.324319	MSTRG.359160	MSTRG.402458	MSTRG.69787
MSTRG.292757	MSTRG.324585	MSTRG.359183	MSTRG.403199	MSTRG.70921
MSTRG.292900	MSTRG.324976	MSTRG.359818	MSTRG.404999	MSTRG.71194
MSTRG.29336	MSTRG.325422	MSTRG.359833	MSTRG.405170	MSTRG.72406
MSTRG.294003	MSTRG.326350	MSTRG.360021	MSTRG.406899	MSTRG.72413
MSTRG.294115	MSTRG.326431	MSTRG.360270	MSTRG.4069	MSTRG.73146
MSTRG.294170	MSTRG.327229	MSTRG.360700	MSTRG.407190	MSTRG.73546
MSTRG.294392	MSTRG.328215	MSTRG.360746	MSTRG.408414	MSTRG.73874
MSTRG.294580	MSTRG.328697	MSTRG.361159	MSTRG.408432	MSTRG.73899
MSTRG.294640	MSTRG.329163	MSTRG.361459	MSTRG.408503	MSTRG.73917
MSTRG.2947	MSTRG.329254	MSTRG.361554	MSTRG.40936	MSTRG.74017
MSTRG.294817	MSTRG.329356	MSTRG.361594	MSTRG.410024	MSTRG.74105
MSTRG.294998	MSTRG.32976	MSTRG.361671	MSTRG.41066	MSTRG.74461
MSTRG.295029	MSTRG.329845	MSTRG.361834	MSTRG.41097	MSTRG.74495
MSTRG.295158	MSTRG.329986	MSTRG.36300	MSTRG.41114	MSTRG.74704
MSTRG.295201	MSTRG.330404	MSTRG.363096	MSTRG.412214	MSTRG.74935
MSTRG.295234	MSTRG.330516	MSTRG.363345	MSTRG.414137	MSTRG.74948
MSTRG.295264	MSTRG.330854	MSTRG.363477	MSTRG.4147	MSTRG.74969
MSTRG.295426	MSTRG.331005	MSTRG.363913	MSTRG.41577	MSTRG.75388
MSTRG.29546	MSTRG.331080	MSTRG.364433	MSTRG.4180	MSTRG.75478
MSTRG.295586	MSTRG.331578	MSTRG.364731	MSTRG.41847	MSTRG.7557
MSTRG.296289	MSTRG.332077	MSTRG.364840	MSTRG.42826	MSTRG.75588
MSTRG.296782	MSTRG.333257	MSTRG.364918	MSTRG.43462	MSTRG.75717
MSTRG.297273	MSTRG.333724	MSTRG.365423	MSTRG.43610	MSTRG.75879
MSTRG.297473	MSTRG.334038	MSTRG.365563	MSTRG.43617	MSTRG.75947
MSTRG.297681	MSTRG.334632	MSTRG.365755	MSTRG.44335	MSTRG.76971
MSTRG.298113	MSTRG.334829	MSTRG.366603	MSTRG.45292	MSTRG.77816
MSTRG.298873	MSTRG.334855	MSTRG.366613	MSTRG.4589	MSTRG.77919
MSTRG.299057	MSTRG.335039	MSTRG.367127	MSTRG.46113	MSTRG.786
MSTRG.299320	MSTRG.335044	MSTRG.367131	MSTRG.46157	MSTRG.78875
MSTRG.299581	MSTRG.335457	MSTRG.367434	MSTRG.46284	MSTRG.79545
MSTRG.299896	MSTRG.335554	MSTRG.367706	MSTRG.46616	MSTRG.79553
MSTRG.300226	MSTRG.335806	MSTRG.367806	MSTRG.47201	MSTRG.80015
MSTRG.300597	MSTRG.336041	MSTRG.368573	MSTRG.47912	MSTRG.80610
MSTRG.30110	MSTRG.336209	MSTRG.36877	MSTRG.48127	MSTRG.80636
MSTRG.301420	MSTRG.336318	MSTRG.369074	MSTRG.48198	MSTRG.80786
MSTRG.301767	MSTRG.33658	MSTRG.369742	MSTRG.48200	MSTRG.80911
MSTRG.301946	MSTRG.337432	MSTRG.369891	MSTRG.48243	MSTRG.81147
MSTRG.302050	MSTRG.33771	MSTRG.370526	MSTRG.48463	MSTRG.81369
MSTRG.302133	MSTRG.337744	MSTRG.371151	MSTRG.48519	MSTRG.81551
MSTRG.302201	MSTRG.337847	MSTRG.371398	MSTRG.48762	MSTRG.81678

MSTRG.302685	MSTRG.338554	MSTRG.371883	MSTRG.49225	MSTRG.82141
MSTRG.302936	MSTRG.338777	MSTRG.372526	MSTRG.51729	MSTRG.82147
MSTRG.303679	MSTRG.338990	MSTRG.373086	MSTRG.51952	MSTRG.82152
MSTRG.303907	MSTRG.338999	MSTRG.373737	MSTRG.52129	MSTRG.82237
MSTRG.304085	MSTRG.341372	MSTRG.373804	MSTRG.52230	MSTRG.82459
MSTRG.304534	MSTRG.341416	MSTRG.373890	MSTRG.52399	MSTRG.83382
MSTRG.304945	MSTRG.342001	MSTRG.374129	MSTRG.52907	MSTRG.83417
MSTRG.305239	MSTRG.342133	MSTRG.374211	MSTRG.53253	MSTRG.83756
MSTRG.305502	MSTRG.342227	MSTRG.374262	MSTRG.53288	MSTRG.86039
MSTRG.305973	MSTRG.342359	MSTRG.374421	MSTRG.53965	MSTRG.86275
MSTRG.306243	MSTRG.343353	MSTRG.374500	MSTRG.54163	MSTRG.87040
MSTRG.306269	MSTRG.343422	MSTRG.374928	MSTRG.54344	MSTRG.87593
MSTRG.307170	MSTRG.34364	MSTRG.375065	MSTRG.54446	MSTRG.87773
MSTRG.307530	MSTRG.343902	MSTRG.375268	MSTRG.54455	MSTRG.87838
MSTRG.308635	MSTRG.345124	MSTRG.376026	MSTRG.54473	MSTRG.88050
MSTRG.308767	MSTRG.345133	MSTRG.376178	MSTRG.54834	MSTRG.88178
MSTRG.309241	MSTRG.345200	MSTRG.376545	MSTRG.55114	MSTRG.88973
MSTRG.309561	MSTRG.345465	MSTRG.376569	MSTRG.55280	MSTRG.89051
MSTRG.309740	MSTRG.345697	MSTRG.377439	MSTRG.55709	MSTRG.89057
MSTRG.309896	MSTRG.346875	MSTRG.377495	MSTRG.56023	MSTRG.8911
MSTRG.311216	MSTRG.34796	MSTRG.37785	MSTRG.56123	MSTRG.90114
MSTRG.311276	MSTRG.348008	MSTRG.378241	MSTRG.5620	MSTRG.91060
MSTRG.311280	MSTRG.348300	MSTRG.378476	MSTRG.56400	MSTRG.91266
MSTRG.311391	MSTRG.348912	MSTRG.378567	MSTRG.5643	MSTRG.92461
MSTRG.311760	MSTRG.349806	MSTRG.380070	MSTRG.56523	MSTRG.92647
MSTRG.311820	MSTRG.349824	MSTRG.380309	MSTRG.57184	MSTRG.92870
MSTRG.312095	MSTRG.350000	MSTRG.381013	MSTRG.57315	MSTRG.9290
MSTRG.312124	MSTRG.350064	MSTRG.381875	MSTRG.57803	MSTRG.93038
MSTRG.31242	MSTRG.350114	MSTRG.382139	MSTRG.58661	MSTRG.93076
MSTRG.312936	MSTRG.350142	MSTRG.382158	MSTRG.58829	MSTRG.93230
MSTRG.313217	MSTRG.350191	MSTRG.382447	MSTRG.59394	MSTRG.93324
MSTRG.313703	MSTRG.350286	MSTRG.382841	MSTRG.59472	MSTRG.93474
MSTRG.313747	MSTRG.350324	MSTRG.383538	MSTRG.595	MSTRG.93559
MSTRG.313817	MSTRG.350799	MSTRG.384638	MSTRG.59716	MSTRG.9499
MSTRG.313819	MSTRG.35096	MSTRG.384977	MSTRG.59800	MSTRG.95241
MSTRG.313908	MSTRG.351348	MSTRG.385018	MSTRG.60463	MSTRG.96149
MSTRG.314603	MSTRG.351359	MSTRG.386027	MSTRG.60909	MSTRG.9634
MSTRG.314974	MSTRG.351581	MSTRG.386204	MSTRG.60954	MSTRG.96965
MSTRG.315452	MSTRG.351605	MSTRG.386717	MSTRG.60977	MSTRG.97206
MSTRG.315622	MSTRG.351636	MSTRG.387126	MSTRG.61786	MSTRG.97415
MSTRG.316301	MSTRG.35167	MSTRG.387773	MSTRG.61870	MSTRG.97497
MSTRG.316357	MSTRG.351885	MSTRG.387787	MSTRG.62475	MSTRG.98480
MSTRG.316501	MSTRG.352497	MSTRG.388992	MSTRG.62933	MSTRG.98486
MSTRG.316503	MSTRG.352595	MSTRG.388995	MSTRG.63033	MSTRG.98521
MSTRG.316987	MSTRG.352677	MSTRG.389238	MSTRG.63168	MSTRG.98855
MSTRG.317741	MSTRG.353776	MSTRG.38938	MSTRG.64334	MSTRG.98954
MSTRG.317827	MSTRG.353963	MSTRG.389732	MSTRG.65717	MSTRG.99004
MSTRG.318146	MSTRG.354113	MSTRG.390239	MSTRG.65827	MSTRG.99352
MSTRG.318548	MSTRG.354195	MSTRG.391286	MSTRG.6583	MSTRG.99580
